# Enrichment and physiological characterization of a novel comammox *Nitrospira* indicates ammonium inhibition of complete nitrification

**DOI:** 10.1038/s41396-020-00827-4

**Published:** 2020-11-13

**Authors:** Dimitra Sakoula, Hanna Koch, Jeroen Frank, Mike S. M. Jetten, Maartje A. H. J. van Kessel, Sebastian Lücker

**Affiliations:** 1 Department of Microbiology, IWWR, Radboud University, Heyendaalseweg 135, 6525 AJ Nijmegen, the Netherlands; 2 Soehngen Institute of Anaerobic Microbiology, Radboud University, Heyendaalseweg 135, 6525 AJ Nijmegen, the Netherlands; 3 Present Address: Division of Microbial Ecology, Center for Microbiology and Environmental Systems Science, University of Vienna, Althanstraße 14, 1090 Vienna, Austria

**Keywords:** Bacterial genomics, Environmental microbiology, Bacterial physiology

## Abstract

The recent discovery of bacteria within the genus *Nitrospira* capable of complete ammonia oxidation (comammox) demonstrated that the sequential oxidation of ammonia to nitrate via nitrite can also be performed within a single bacterial cell. Although comammox *Nitrospira* exhibit a wide distribution in natural and engineered ecosystems, information on their physiological properties is scarce due to the limited number of cultured representatives. Additionally, most available genomic information is derived from metagenomic sequencing and high-quality genomes of *Nitrospira* in general are limited. In this study, we obtained a high (90%) enrichment of a novel comammox species, tentatively named “*Candidatus* Nitrospira kreftii”, and performed a detailed genomic and physiological characterization. The complete genome of “*Ca*. N. kreftii” allowed reconstruction of its basic metabolic traits. Similar to *Nitrospira inopinata*, the enrichment culture exhibited a very high ammonia affinity (K_m(app)_NH3_ ≈ 0.040 ± 0.01 µM), but a higher nitrite affinity (K_m(app)_NO2_- = 12.5 ± 4.0 µM), indicating an adaptation to highly oligotrophic environments. Furthermore, we observed partial inhibition of ammonia oxidation at ammonium concentrations as low as 25 µM. This inhibition of “*Ca*. N. kreftii” indicates that differences in ammonium tolerance rather than affinity could potentially be a niche determining factor for different comammox *Nitrospira*.

## Introduction

Nitrification, the biological oxidation of ammonia to nitrate via nitrite, is a critical process within the global biogeochemical nitrogen cycle. The nitrification process is of great biotechnological relevance since it fuels the reductive part of the nitrogen cycle and is widely employed in drinking and wastewater treatment systems for the efficient removal of excess ammonium. Traditionally, nitrification was considered to be a two-step process catalyzed by two functionally distinct microbial guilds. According to this paradigm, ammonia-oxidizing prokaryotes first oxidize ammonia to nitrite and subsequently nitrite-oxidizing bacteria (NOB) are responsible for the conversion of nitrite to nitrate. While this dogma has been challenged by the theoretical prediction of complete ammonia oxidation (comammox) [1, 2], it was the discovery of comammox *Nitrospira* that has drastically altered our perception of nitrification [3–5].

All comammox organisms described to date are affiliated with *Nitrospira* sublineage II and can be further divided into clade A and B based on phylogeny of the ammonia monooxygenase, the enzyme catalyzing the first step of ammonia oxidation [4]. Comammox *Nitrospira* were identified mainly via metagenomic sequencing in various natural and engineered ecosystems, indicating their widespread occurrence and key role in nitrogen cycling [6–15]. This ubiquitous abundance of comammox *Nitrospira* has raised many questions regarding their ecophysiology and potential biotechnological applicability. In order to provide the necessary answers, in-depth understanding of the comammox physiology is required. So far, the sole physiological data available was obtained from *Nitrospira inopinata*, the only existing pure culture of a comammox bacterium [4]. The extremely low apparent half saturation constant (K_m(app)_) for ammonia and the high growth yield reported for *N. inopinata* indicate an adaptation to nutrient-limited environments [16] and corroborate the predicted comammox lifestyle [1].

A general adaptation of comammox *Nitrospira* to oligotrophic environments is suggested by their presence mainly in ecosystems with low ammonium loads. However, limited physiological data can highly bias our perception of the ecophysiology of certain microbial groups and kinetic parameters might vary between different comammox species. This was for instance recently observed for ammonia-oxidizing archaea (AOA) and bacteria (AOB), where especially terrestrial AOA were found to have lower ammonia affinities than previously assumed based on the extremely low K_m_ reported for the marine AOA *Nitrosopumilus maritimus* [16, 17]. For comammox *Nitrospira*, though, the lack of pure cultures or high enrichments hampers the thorough understanding of the ecophysiology of these intriguing microorganisms.

In this study, we describe the enrichment of a novel comammox *Nitrospira* species in a continuous membrane bioreactor system and provide genome-derived insights into its metabolic potential. Furthermore, we report the ammonia- and nitrite-oxidation kinetics of this comammox organism, including an apparent inhibition by ammonium concentrations as low as 25 µM, findings that provide crucial insights into the potential niche partitioning factors of different comammox *Nitrospira*.

## Materials and methods

### Enrichment and reactor operation

A 7 L continuous membrane bioreactor (Applikon, Delft, The Netherlands), with a working volume of 5 L was inoculated with biomass from a hypoxic (≤3.1 µM O_2_) enrichment culture, that contained two distinct comammox *Nitrospira* species [3]. At the start of the system’s operation, 300 mL of the previously described comammox enrichment culture were resuspended in 4.7 L of substrate-free mineral salts medium (for composition see below). The bioreactor was operated for 39 months at room temperature (RT) with moderate stirring (200 rpm). The pH of the culture was constantly monitored by a pH electrode connected to an ADI1020 biocontroller (Applikon, Delft, The Netherlands) and maintained at 7.5 by the automatic supply of a 1 M KHCO_3_ solution. Dissolved oxygen was kept at 50% saturation by providing 10 mL/min of a mixture of Argon/CO_2_ (95%/5% v/v) and air through a metal tube equipped with a porous sparger. The gas ratio was manually adjusted through the system’s operation period in order to maintain 50% oxygen saturation in the system. The level of the system was controlled by the ADI1020 biocontroller and maintained at 5 L working volume by removal of effluent via the membrane filtration system, ensuring biomass retention in the system. Following inoculation, 1 L of sterile NOB mineral salts medium [18] was supplied to the reactor per day. The medium was supplemented with 1 mL of a trace element stock solution composed of NTA (15 g/L), ZnSO_4_⋅7H_2_O (0.43 g/L), CoCl_2_⋅6H_2_O (0.24 g/L), MnCl_2_⋅4H_2_O (0.99 g/L), CuSO_4_⋅5H_2_O (0.25 g/L), Na_2_MoO_4_⋅2H_2_O (0.22 g/L), NiCl_2_⋅6H_2_O (0.19 g/L), NaSeO_4_·10H_2_O (0.021 g/L), H_3_BO_4_ (0.014 g/L), CeCl·6H_2_O (0.24 g/L) and 1 ml of an iron stock solution composed of NTA (10 g/L) and FeSO_4_ (5 g/L). Initially, ammonium, nitrite and nitrate (80/0/50 µM NH_4_Cl/NaNO_2_/NaNO_3_, respectively, increased to 250/20/500 on day 27) were supplied via the medium. After 2 months of operation, ammonium was supplied as the sole substrate and the concentration in the medium was slowly increased from initially 250 µM NH_4_Cl (day 60) to a final concentration of 2.5 mM (day 453; Fig. S1). After 15 months of operation a bleed was installed in addition to the level-controlled media removal over the membrane, which removed 100 to 300 mL biomass per day, depending on the density and activity of the biomass. Liquid samples from the bioreactor were collected regularly for the determination of ammonium, nitrite and nitrate concentrations.

### Analytical methods

Ammonium concentrations were measured colorimetrically via a modified orthophatal-dialdehyde assay (detection limit 10 µM) [19]. Nitrite concentrations were determined by the sulfanilamide reaction (detection limit 5 µM) [20]. Nitrate was measured by converting it into nitric oxide at 95 °C using a saturated solution of VCl_3_ in HCl, which was subsequently measured using a nitric oxide analyzer (detection limit 1 µM; NOA280i, GE Analytical Instruments, Manchester, UK). Protein extraction and determination were performed using the B-PER™ Bacterial Protein Extraction Reagent and Pierce™ BCA Protein Assay Kit (Thermo Fisher Scientific, Waltham, MA, USA), respectively.

### Fluorescence in situ hybridization

Biomass samples were fixed using a 3% (v/v) paraformaldehyde (PFA) solution for 30 min at RT. Fluorescence in situ hybridization (FISH) was performed as described elsewhere [3] using 16S rRNA-targeted oligonucleotide probes (Table S1) that were fluorescently labeled with Fluoresceine or the cyanine dyes Cy3 or Cy5. After hybridization, slides were dried and embedded in Vectashield mounting solution (Vector Laboratories Inc., Burlingame, CA, USA). For image acquisition a Leica TCS SP8x confocal laser scanning microscope (Leica Microsystems, Wetzlar, Germany) equipped with a pulsed white light laser and a 405 nm diode was used. In order to quantify the total *Nitrospira* biovolume in the enrichment culture fixed biomass was hybridized with the genus and phylum-specific probes Ntspa662 and Ntspa712 (labeled in the same color), respectively, and EUB338mix (Table S1). Subsequently, at least 15 image pairs were recorded at random fields of view. The images were imported into the image analysis software *daime* [21] and analyzed as described elsewhere [22]. Similarly, the biovolumes of sublineage I and II *Nitrospira* were quantified using the probes Ntspa1431 and Ntspa1151, respectively, in combination with a mix of Ntspa662 and Ntspa712 (Table S1).

### DNA extraction

After 17 and 39 months of enrichment, DNA was extracted from 50 ml of culture using the PowerSoil DNA Isolation Kit (MO BIO Laboratories Inc., Carlsbad, CA, USA) and a CTAB-based DNA extraction method (17 months sample) [23] or the DNeasy Blood & Tissue Kit (39 months sample; Qiagen, Hilden, Germany). Concentration and quality of the obtained DNA were checked using a Qubit™ dsDNA HS Assay Kit (Thermo Fisher Scientific, Waltham, MA, USA) on a Qubit Fluorometer (Thermo Fisher Scientific, Waltham, MA, USA) and a NanoDrop™ 1000 Spectrophotometer (Thermo Fisher Scientific), respectively.

### Metagenome sequencing and analysis

Metagenome sequencing was performed using an Illumina MiSeq benchtop DNA sequencer (Illumina Inc., San Diego, California USA). Genomic sequencing libraries were prepared using the Nextera XT Kit (Illumina Inc., San Diego, California U.S.A.) following the manufacturer’s instructions, using 1 ng of input DNA normalized to a 0.2 ng/µl concentration. The MiSeq Reagent Kit v3 (2 × 300 bp) (Illumina Inc., San Diego, California USA) was used for sequencing according to manufacturer’s recommendations.

Sequencing adapter removal, quality-trimming and contaminant filtering of Illumina paired-end sequencing reads was performed using BBDuk version 37.76 from the BBTools package (https://jgi.doe.gov/data-and-tools/bbtools). Processed reads for all samples were co-assembled using metaSPAdes v3.11.1 [24] with default parameters. MetaSPAdes iteratively assembled the metagenome using k-mer lengths 21, 33, 55, 77, 99 and 127. Reads were mapped back to the assembled metagenome for each sample separately using Burrows-Wheeler Aligner (BWA) v0.7.17 [25], employing the “mem” algorithm. The sequence mapping files were processed using SAMtools v1.6 [26]. Metagenome binning was performed for contigs ≥2000 bp. To optimize binning results, five binning algorithms were used: BinSanity v0.2.6.1 [27], COCACOLA [28], CONCOCT v0.4.1 [29], MaxBin 2.0 v2.2.4 [30] and MetaBAT 2 v2.12.1 [31]. To obtain the final bins, the five bin sets subsequently were supplied to DAS Tool v1.0 [32] for consensus binning. The quality of the genome bins was assessed through a single-copy marker gene analysis using CheckM v1.0.7 [33]. The GTDB-Tk software was used for taxonomic classifications to the obtained bins [34, 35]. Only *Nitrospira* bins with estimated completeness ≥90% and contamination ≤10% were included in subsequent analyses.

### Nanopore sequencing and assembly of “Ca. N. kreftii”

To assemble the complete genome of the dominant *Nitrospira* species, single-molecule long-read data was obtained after 17 months of enrichment using the Oxford Nanopore MinION platform (Oxford Nanopore Technologies, Oxford, UK). Genomic DNA was extracted by using the CTAB-based protocol as described above and prepared for sequencing using the Ligation Sequencing Kit 1D (SQK-LSK108, Oxford Nanopore Technologies) according to the manufacturer’s instructions. Adapter-ligated DNA was cleaned by adding 0.8 volumes of AMPure XP beads (Beckman Coulter Inc., Brea, CA, USA). Sequencing libraries were loaded on a SpotOn Flow Cell (FLO-MIN106 R9.4, Oxford Nanopore Technologies) using the Library Loading Beads Kit (EXP-LLB001, Oxford Nanopore Technologies) following manufacturer’s specifications.

Sequencing was performed on the MinION sequencing device with MinKNOW v1.7.10 software using the FLO-MIN106 450 bps protocol. Base calling of the signal data was performed using Guppy v2.3.7 (Oxford Nanopore Technologies) with the flipflop model -c dna_r9.4.1_450bps_flipflop.cfg. Only NanoPore reads with a length ≥700 bp were used for further analyses. The Nanopore reads were assembled de novo using Canu v1.8 [36] with parameters “genomeSize = 50m” and “corOutCoverage = 1000”. Subsequently, all Nanopore reads with a length of ≥700 bp were mapped to the assembly using minimap2 v2.16-r922 [37], followed by building genomic consensus sequences using Racon v1.3.1 [38]. This long read assembly approach resulted in a closed *Nitrospira* genome whose taxonomic classification was confirmed using the classify_wf workflow of GTDB-Tk v0.3.2 [35] with default settings. All trimmed Illumina reads of the 17 months sample were mapped to this complete *Nitrospira* genome using bbmap v37.76 (sourceforge.net/projects/bbmap/) with “minid 0.8”. A hybrid assembly was performed using Unicycler v0.4.4 with the mapped Illumina reads and the NanoPore reads as input and the NanoPore consensus assembly as existing long read assembly. In addition, the chromosomal replication initiator protein, DnaA of *Nitrospira moscoviensis* (ALA56445.1) was used to set *dnaA* as starting gene with the parameters “start_gene_id 60” and “start_gene_cov 80”.

### Phylogenomic analysis and genome annotation

Reference genomes that were downloaded from the NCBI GenBank database (13/05/2019) and the *Nitrospira* metagenome-assembled genomes (MAGs) retrieved in this study were dereplicated using dRep [32] with default parameters filtering for an estimated completeness ≥90% and contamination ≤10%. The UBCG pipeline [39] was used for phylogenomic analysis of the obtained *Nitrospira* MAGs and 34 publicly available, high-quality genomes of sublineage I and II *Nitrospira*. UBCG was used to identify and extract 91 bacterial single copy core genes from all genomes. After alignment in UBCG with default parameters, a maximum likelihood phylogenetic tree was calculated based on the concatenated nucleotide alignment using RAxML v8.2.12 [40] on the CIPRES science gateway [41] with the GTR substitution and GAMMA rate heterogeneity models and 100 bootstrap iterations. Two *Leptospirillum* genomes were used as outgroup. Average nucleotide identity (ANI) values of the genomes were calculated using the OrthoANIu tool [42].

All CDS of the high-quality draft genomes of *Nitrospira* including the complete genome of “*Ca*. N. kreftii” were automatically predicted and annotated using a modified version Prokka [43] that performs a BLASTp search of all CDS against the NCBI RefSeq non-redundant protein database (version 92). Homologous proteins in these MAGs and in selected *Nitrospira* genomes were identified by reciprocal best BLAST. Only BLAST hits with an e-value ≤1e-06, amino acid similarity ≥35% and minimum alignment coverage of 80% were considered as homologous proteins. In addition, the automatic annotation of selected genes of “*Ca*. N. kreftii” was confirmed using the annotation platform Genoscope [44]. The visualization tool Circos v0.69-6 [45] was used to generate a whole genome map of “*Ca*. N. kreftii”.

### Adaptation to increased substrate concentrations in batch culturing conditions

Biomass (1 L) from the enrichment culture was washed twice in sterile NOB medium by centrifugation (1500 × *g*, 2 min) and subsequent resuspension in the same volume of 0.01 M HEPES buffered (pH 7.5) sterile NOB medium containing 0.1 mM KHCO_3_ supplemented with 1 mM NH_4_Cl. The culture was incubated in the dark for 30 days (RT, 150 rpm, rotary shaker). Upon full ammonium consumption, substrate was replenished (approximately every 7 days). At the end of the adaptation period, a total of 4 mM of NH_4_Cl were completely and stoichiometrically oxidized to nitrate in the batch culture. In order to avoid potential inhibition due to nitrate accumulation, the culture was washed after 15 days of incubation. During the washing, the complete culture was centrifuged (1500 × *g*, 2 min) and the medium was exchanged with 1 L of fresh HEPES buffered sterile NOB medium.

### Substrate-dependent oxygen uptake rate measurements

After 39 months of enrichment, the activity of the culture was determined by microrespirometry. Biomass from 20 mL of the bioreactor or the batch cultures was harvested, washed twice by centrifugation (1500 × *g*, 2 min) and finally resuspended in 2 mL of 0.01 M HEPES buffered sterile NOB medium containing 0.1 mM KHCO_3_. Oxygen consumption rates were measured at 25 °C using a RC-350 respiration chamber (Warner Instruments LLC, Hamden, USA), equipped with an oxygen sensor (Model 1302, Warner Instruments LLC, Hamden, USA) and connected to a picoammeter PA2000 (Unisense, Aarhus, Denmark). NH_4_Cl or NaNO_2_ were injected from concentrated stock solutions (1 mM) into the reaction vessel. At the end of the measurements, biomass was harvested for protein and floc size determination. Concentrations of ammonium, nitrite and nitrate were determined in the supernatant as described above.

### Calculation of kinetic parameters

The kinetic constants of the enrichment culture were estimated from oxygen consumption measurements using substrate:oxygen consumption stoichiometries of 1:2 and 2:1 for ammonia and nitrite oxidation, respectively. Measurements were corrected for background respiration, which were determined from oxygen uptake rates prior to substrate addition.

Ammonia oxidation by “*Ca*. N. kreftii” was best described by the Haldane substrate inhibition model (Eq. (1)) and K_i_ values were calculated based on fitting of the data to this model. Due to the overestimation of the K_m(app)_ and V_max_ values by the inhibition model, these were obtained by fitting the experimental data obtained for non-inhibitory ammonium concentrations to a Michaelis–Menten model (Eq. (2)), which was also employed to calculate K_m(app)_ and V_max_ for nitrite oxidation.

1 $$V = \frac{{V_{\max }[S]}}{{K_{m(app)} + \left[ S \right] + \frac{{\left[ S \right]^2}}{{K_i}}}}$$

2 $$V = \frac{{V_{\max }\left[ S \right]}}{{K_{m\left( {app} \right)} + \left[ S \right]}}$$

with V representing the observed oxidation rate, V_max_ the maximum rate (in µM h^−1^), K_m(app)_ the apparent Michaelis–Menten half saturation constant, K_i_ the inhibition parameter that is equal to the maximum substrate concentration that produces a rate of 1/2 *V*_max_, and [S] the substrate concentration (in µM).

### Floc size determination and statistical analyses

A representative biomass sample form the bioreactor enrichment culture, as well as the collected biomass at the end of the microrespirometry experiments (see below), was used for the determination of the average floc size (area) using image analysis. Microscopic images were acquired using a Zeiss Axioplan 2 (Carl Zeiss AG, Oberkochen, Germany) light microscope. Floc area was calculated manually using the software platform ImageJ [46].

The Pearson’s coefficient (*r*), as well as the significance level (*p* value, *p*) of the correlations between floc size and the corresponding apparent half saturation affinity constants for ammonium and nitrite were calculated using the ‘rstatix’ package (v.0.6.0) [47] in *R* (v.3.6.2) [48]. The degrees of freedom (corresponding to the number of data points -2) used to calculate *r* are indicated in brackets. All values are reported according to the APA guidelines.

## Results

### Enrichment of comammox Nitrospira

A continuous laboratory-scale membrane bioreactor was used for the enrichment of comammox *Nitrospira*. The bioreactor was inoculated with biomass from an enrichment culture containing two identified comammox *Nitrospira* species (“*Ca*. Nitrospira nitrosa” and “*Ca*. Nitrospira nitrificans”), which constituted together ~15% of the microbial community [3]. Since comammox bacteria are speculated to thrive under highly limiting substrate concentrations, medium amended with low ammonium concentrations was supplied to the system. Additionally, the system was operated at 50% oxygen saturation. The total ammonium load of the system was, based on the consumption rate and biomass concentration in the culture, gradually increased from initially 0.016 to finally 2.5 mmol day^−1^ (Fig. S1A) and was stoichiometrically oxidized to nitrate (Fig. S1B). Concentrations of ammonium and nitrite in the bioreactor always remained below the detection limit (10 µM; Fig. S1B).

**Fig. 1:**
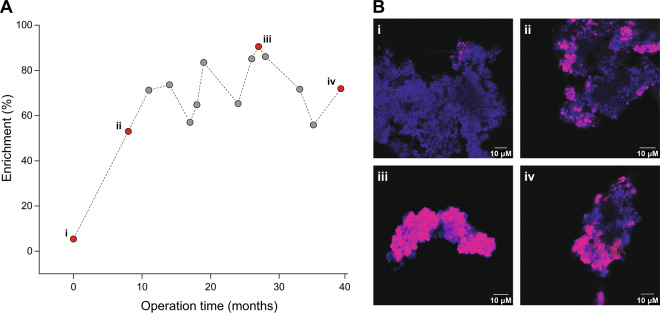
Enrichment of *Nitrospira* bacteria in the bioreactor system. **a** Relative abundance of *Nitrospira* bacteria over the enrichment period. **b** Representative fluorescent micrographs of biomass samples obtained from the enrichment culture throughout the enrichment period. (i) Starting inoculum of the bioreactor and biomass sampled after (ii) 8, (iii) 26 and (iv) 39 months of enrichment. Cells are stained using FISH probes for all bacteria (EUB338mix, blue) and *Nitrospira*-specific probes (Ntspa712 and Ntspa662, red).

After 27 months of operation, *Nitrospira* bacteria were present in suspended flocs of an average area of 9.8 ± 4 µm^2^ (range 0.3–12.5 µm^2^) and constituted ~90% of the total microbial community in the culture, as revealed by quantitative FISH (Fig. 1, Table S2). Subsequently, the relative abundance of *Nitrospira* dropped due to a malfunction of our sterilization system that resulted in the introduction of heterotrophic bacteria into the bioreactor. Despite this temporary reduction in the degree of enrichment, bacteria belonging to the genus *Nitrospira* dominated the microbial community over the whole 39 months of operation.

Quantification of the relative abundances of *Nitrospira* affiliated with sublineages I and II revealed that up to 95 ± 6% of the total *Nitrospira* population consisted of sublineage II *Nitrospira*, while sublineage I never constituted more than 3.1 ± 1% (Fig. S2). FISH with probes targeting the known AOA or betaproteobacterial AOB indicated their absence from the culture at all time points analyzed (data not shown), as was already the case for the inoculum [3].

### Metagenomic retrieval of a novel clade A comammox Nitrospira

**Fig. 2:**
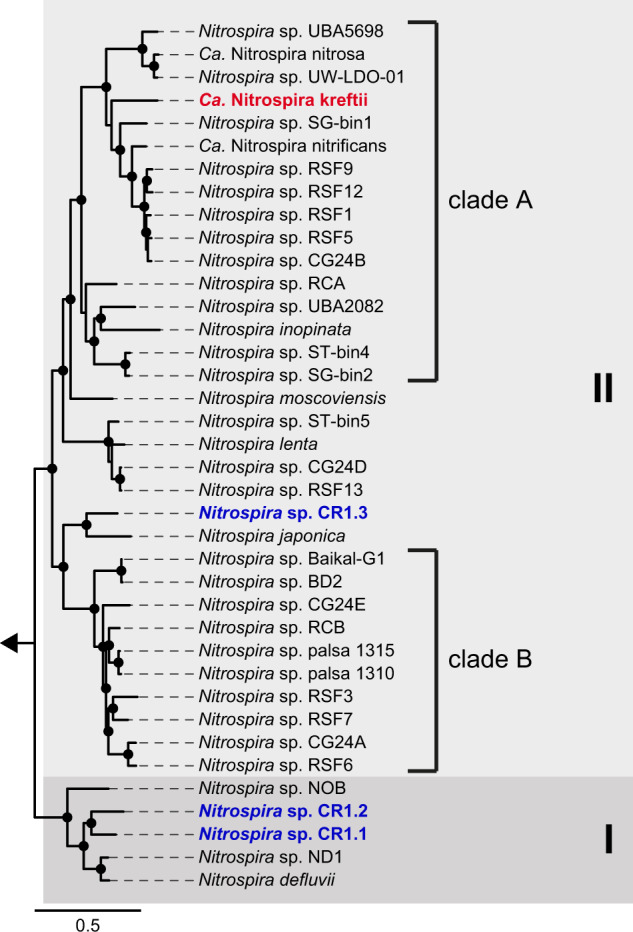
Phylogenomic analysis of the retrieved *Nitrospira* MAGs and representative sublineages I and II *Nitrospira*. Sublineages are indicated by shaded boxes and labeled with roman numerals, comammox clades are designated by square brackets. Bootstrap support values ≥99% are indicated by black circles. The arrow indicates the position of the outgroup, which consisted of two *Leptospirillum* species. The scale bar corresponds to 50% estimated sequence divergence.

Metagenome sequencing in combination with de novo assembly and consensus binning of the microbial community present in the bioreactor enrichment after 17 months of operation resulted in the recovery of 28 metagenome-assembled genomes (MAGs) of medium or high quality (completeness ≥75% or ≥90%, respectively, and contamination ≤10%; Dataset S1). Of these, four high-quality MAGs were affiliated with the genus *Nitrospira*. The number of reads mapped to these *Nitrospira* MAGs corresponded to 36% of the total reads and total coverage data suggested that one *Nitrospira* MAG dominated the microbial community in the bioreactor system (Dataset S1). Phylogenomic analysis revealed that this MAG belongs to a novel clade A comammox *Nitrospira* (Figs. 2 and S3). The remaining *Nitrospira*-like MAGs clustered with canonical nitrite-oxidizing *Nitrospira* within sublineage I (2 MAGs; *Nitrospira* spp. CR1.1 and CR1.2) and sublineage II (1 MAG; *Nitrospira* sp. CR1.3; Figs. 2 and S3). In combination with the lack of key genes for ammonia oxidation (Fig. S4), this phylogenetic affiliation strongly indicated that these *Nitrospira* were canonical nitrite oxidizers.

A hybrid assembly approach for scaffolding the Illumina assembly with long Nanopore reads allowed the retrieval of the complete genome of the dominant *Nitrospira* MAG, with a total size of 4.13 Gb and an overall G+C content of 54.5%. The average nucleotide identities (ANI) of this genome to 34 high-quality genomes of sublineage I and II *Nitrospira* available at the time of study (May 13, 2019) are ≤77% (Fig. S3), which is below the species cutoff of 95% [49]. Together with the phylogenetic distance in the phylogenomic analysis (Fig. 2), this classifies it as a novel clade A comammox *Nitrospira*, which we tentatively named ‘*Candidatus* Nitrospira kreftii’. Intriguingly, this novel species apparently outcompeted the two comammox species detected in the source enrichment [3], presumably due to the changes in substrate and oxygen concentrations supplied to the culture (see above).

Resequencing after additional 22 months of enrichment indicated a clear decrease in diversity for both *Nitrospira* and the overall microbial community. More specifically, after a total of 39 months of enrichment, we retrieved 9 medium and 7 high-quality MAGs, out of which “*Ca*. N. kreftii” was the only MAG affiliated with sublineage II of the genus *Nitrospira* (Dataset S2). The metagenome contained one additional MAG (*Nitrospira* sp. CR2.1) representing a canonical sublineage I *Nitrospira*, which was highly similar (96% ANI) to the *Nitrospira* sp. CR1.2 MAG obtained from the 17-month sample. However, this MAG showed >10% estimated contamination, most likely indicating wrong assignments of contigs belonging to *Nitrospira* sp. CR1.1 into this genome bin. Putatively heterotrophic microorganisms accounted for the rest of the microbial community present in the system (Dataset S2). No canonical ammonia-oxidizing prokaryotes were identified in the metagenomic datasets, confirming that “*Ca*. N. kreftii” was indeed the only ammonia oxidizer in the system (Datasets S1 and S2).

### Metabolic potential of “Ca. N. kreftii”

**Fig. 3:**
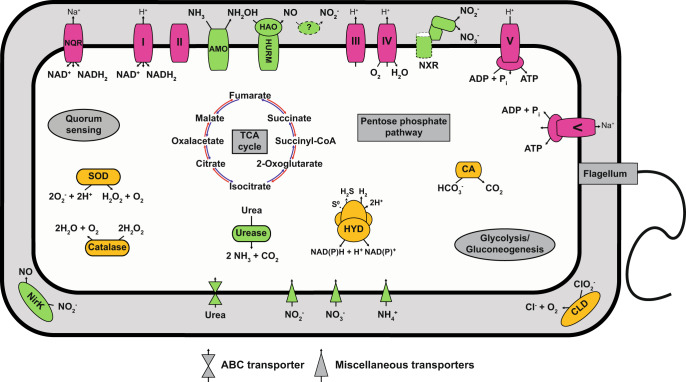
Cell metabolic cartoon of “*Ca*. N. kreftii”. AMO ammonia monooxygenase, HAO hydroxylamine dehydrogenase, NXR nitrite oxidoreductase, HYD group 3b [NiFe]-hydrogenase, CA carbonic anhydrase, CLD chlorite dismutase, SOD superoxide dismutase, NirK Cu-dependent nitrite reductase, NQR Na^+^-translocating NADH:ubiquinone oxidoreductase. Enzyme complexes of the electron transport chain are labeled by Roman numerals. Dashed lines indicate putative features. The question mark indicates that the exact enzyme catalyzing the nitrite formation from NO remains uncertain.

Analysis of the complete “*Ca*. N. kreftii” genome revealed the presence of all genes for the enzyme complexes involved in complete nitrification (i.e., ammonia monooxygenase (AMO), hydroxylamine dehydrogenase (HAO) and nitrite oxidoreductase (NXR); Fig. 3, Dataset S3). Similar to most comammox *Nitrospira*, the “*Ca*. N. kreftii” genome contained one gene cluster encoding the structural AMO subunits (*amoCAB*), the hydroylamine:ubiquinone reduction module (HURM; consisting of *haoAB* for the HAO structural subunits and *cycAB*, encoding the cytochromes *c*554 and *c*_M_552) as well as genes for the type I cytochrome *c* biosynthesis system. In addition, “*Ca*. N. kreftii” harbors four non-operonal *amoC* copies and an additional *haoA* (Dataset S3). For nitrite oxidation, the genome contains two *nxrAB* gene clusters encoding the alpha and beta subunit of the periplasmic NXR and four non-operonal genes for putative gamma subunits (*nxrC*; Fig. S5). One of these *nxrC* is clustered with a TorD-like chaperone probably involved in insertion of the molybdopterin cofactor into the catalytic NxrA subunits, and a NapG-like ferredoxin as has been described for other *Nitrospira* [50–52]. As found in all *Nitrospira* [53, 54], also “*Ca*. N. kreftii” encodes a copper-containing nitrite reductase (NirK; Dataset S3), the exact function of which however still is unclear [16].

In addition to the enzyme systems for ammonia and nitrite oxidation, “*Ca*. N. kreftii” encodes all complexes of the respiratory chain, the reductive tricarboxylic acid cycle for CO_2_ fixation, and the complete gene repertoire for glycolysis/gluconeogenesis and the oxidative and non-oxidative phases of the pentose phosphate pathway, which all belong to the core metabolism of the genus *Nitrospira*. Notably, the genome of “*Ca*. N. kreftii” encoded an alternate F_1_F_o_-type ATPase (also referred to as Na^+^-translocating N-ATPase; [55]), and an alternative sodium-pumping complex I (Na^+^-NQR; Dataset S3) [56]. These features have been identified in other aerobic and anaerobic ammonia oxidizers, as well as in the nitrite-oxidizing “*Ca*. Nitrospira alkalitolerans”, where they were linked to an adaptation to haloalkaline environments [57–59].

Similar to other clade A comammox *Nitrospira* [3, 16, 60], “*Ca*. N. kreftii” featured a low-affinity Rh-type transporter for ammonium uptake, and possessed a complete operon encoding the structural and accessory urease subunits and a high-affinity urea transporter. This indicates that besides ammonia also urea can be used as source of ammonium for assimilation and to fuel ammonia oxidation under ammonium-limited conditions [3]. While canonical *Nitrospira* including those identified in this study can use nitrite as nitrogen source for assimilation (Fig. S4), no assimilatory nitrite reduction system was identified in the complete genome of “*Ca*. N. kreftii” (Dataset S3), as is the case in all other available comammox genomes [13].

### Kinetic characterization of the enrichment culture

Both FISH and metagenomic sequencing indicated the absence of known canonical ammonia oxidizers in the enrichment culture, and demonstrated “*Ca*. N. kreftii” to be the dominant nitrifier and only comammox *Nitrospira* in the system. Thus, the enrichment culture was used to determine the apparent kinetic parameters of the nitrification reaction by measuring the substrate-dependent oxygen (O_2_) uptake rates using microrespirometry.

**Fig. 4:**
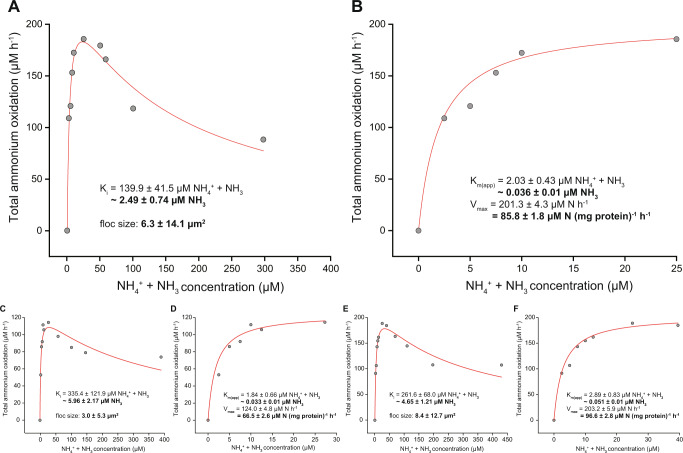
Ammonia oxidation kinetics of the “*Ca*. N. kreftii” enrichment culture. Data from three biological replicates is shown. The red curves indicate the best fit of all data to the substrate inhibition model (**a**, **c**, **e**) and of the data retrieved for non-inhibitory ammonium concentrations in a Michaelis–Menten kinetic equation (**b**, **d**, **f**). The reported standard errors are based on nonlinear regression.

O_2_ consumption immediately increased upon substrate addition and ammonium and O_2_ were consumed in a 1:2 stoichiometry (1:1.96 ± 0.13 mean ± s.d., *n* = 4), as expected for complete nitrification, while only a transient accumulation of low concentrations of nitrite (1–5 µM) was observed. From this data we estimated a mean apparent half-saturation constant (K_m(app)_) of 2.25 ± 0.56 µM total ammonium (NH_4_^+^ + NH_3_), corresponding to K_m(app)_ ≈ 0.040 ± 0.010 µΜ ammonia (*n* = 3; Fig. 4). The mean maximum total ammonium oxidation rate (V_max_) of 83.0 ± 15.2 µmol NH_4_^+^ + NH_3_ (mg protein)^−1^ h^−1^ (*n* = 3) was reached at concentrations as low as 25 µM. Surprisingly, ammonia oxidation by the enrichment culture did not follow typical Michaelis–Menten kinetics and ammonium concentrations >25 µM caused a reduction in V_max_. Consequently, ammonia oxidation in “*Ca*. N. kreftii” was better described using the Haldane substrate inhibition model, which yielded a mean apparent inhibition constant (K_i(app)_) of 245.7 ± 98.7 µM total ammonium, corresponding to K_i(app)_ ≈ 4.37 ± 1.76 µM ammonia (*n* = 3; Fig. 4). However, it should be noted that “*Ca*. N. kreftii” was not completely inhibited by elevated ammonium concentrations, but retained ~50% of V_max_ also at ammonium concentrations up to 450 µM, thus impeding accurate estimations of K_i_.

**Fig. 5:**
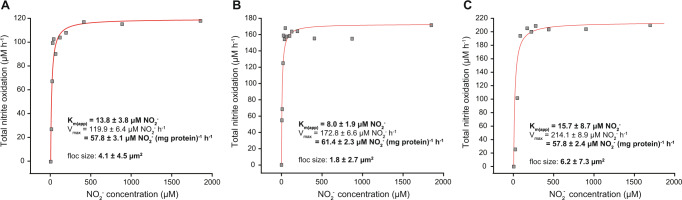
Nitrite oxidation kinetics of the “*Ca*. N. kreftii” enrichment culture. Data from three biological replicates is shown. The red curve indicates the best fit of the data to the Michaelis–Menten kinetic equation. The reported standard errors are based on nonlinear regression.

Contrastingly, nitrite oxidation in the enrichment culture followed typical Michaelis–Menten kinetics. Nitrite was oxidized to nitrate with the expected 2:1 nitrite:oxygen stoichiometry (2:1.04 ± 0.04, *n* = 3) and we determined mean K_m(app)_ and V_max_ values of 12.5 ± 4.0 µM nitrite and 59.0 ± 2.1 µM nitrite (mg protein)^−1^ h^−1^, respectively (*n* = 3; Fig. 5).

**Fig. 6:**
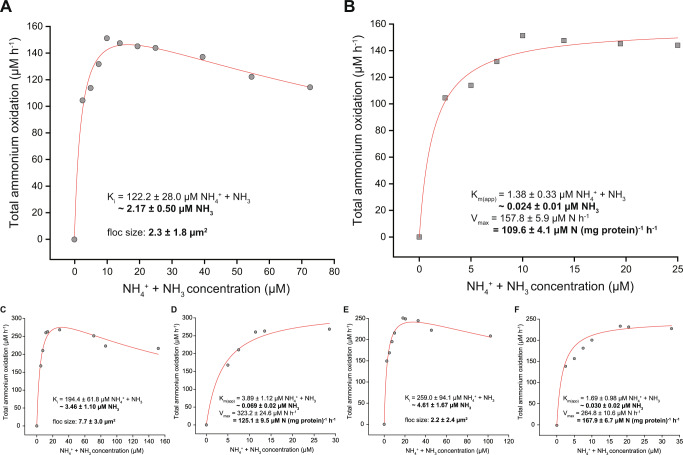
Ammonia oxidation kinetics of the “*Ca*. N. kreftii” enrichment culture adapted to 1 mM ammonium feeding. Data from three biological replicates is shown. The red curves indicate the best fit of all data to the substrate inhibition model (**a**, **c**, **e**) and of the data retrieved for non-inhibitory ammonium concentrations in a Michaelis–Menten kinetic equation (**b**, **d**, **f**). The reported standard errors are based on nonlinear regression.

**Fig. 7:**
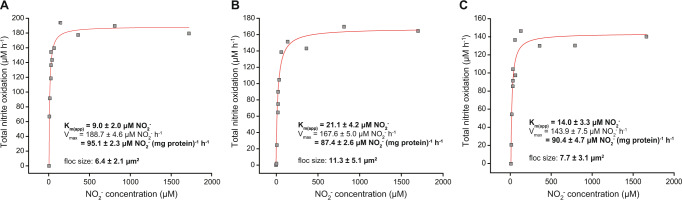
Nitrite oxidation kinetics of the “*Ca*. N. kreftii” enrichment culture adapted to 1 mM ammonium feeding. Data from three biological replicates is shown. The red curve indicates the best fit of the data to the Michaelis–Menten kinetic equation. The reported standard errors are based on nonlinear regression.

For non-planktonic microbial cultures, substrate uptake kinetics are influenced by the size and shape of the microcolonies the microorganism forms, and the thickness of the biofilm or, in case of suspended growth, floc size [61, 62]. The average floc size of the biomass during determination of the ammonia and nitrite oxidation kinetic parameters was 5.5 ± 10.4 µm^2^ and 4.4 ± 6.0 µm^2^, respectively (ranging from 1.8 ± 2.7 to 8.4 ± 12.7 µm^2^). As expected, a positive correlation was observed between the determined ammonia (*r* (4) = 0.84, *p* = 0.038; *n* = 6) and nitrite (*r* (4) = 0.81, *p* = 0.051; *n* = 6) K_m(app)_ values and the degree of biomass aggregation, with larger average floc sizes corresponding to increased K_m(app)_ values (Figs. 4–7).

To exclude that the observed inhibition pattern of ammonia oxidation was due to a potential physiological adaptation of the biomass to the continuous substrate-limited culturing conditions, batch cultures at higher ammonium concentrations (1 mM NH_4_^+^) were initiated. After one month of cultivation with substrate replenishment when ammonium was fully consumed, ammonia and nitrite oxidation kinetics were determined as before. However, also with this high substrate-adapted biomass, a similar inhibition pattern was observed upon addition of ammonium concentrations >25 µM (Fig. 6), whereas the nitrite oxidation kinetics again followed Michaelis–Menten type kinetics (Fig. 7). Fitting of the converted oxygen uptake data to Eqs. (1) and (2) (see Materials and Methods) yielded mean K_m(app)_ (2.32 ± 1.37 µM NH_4_^+^ + NH_3_, corresponding to 0.041 ± 0.024 µΜ NH_3_; 14.7 ± 6.1 µM NO_2_^−^), V_max_ (134.2 ± 30.2 µmol NH_4_^+^ + NH_3_ (mg protein)^−1^ h^−1^; 91.0 ± 3.1 µM NO_2_^−^ (mg protein)^−1^ h^−1^) and K_i(app)_ (191.9 ± 68.5 µM NH_4_^+^ + NH_3_) values for ammonia and nitrite oxidation, respectively, that where comparable to those obtained with the continuous bioreactor culture. Thus, while the slight increment in V_max_ for ammonia and nitrite oxidation, and possibly also the slight reduction in the degree of inhibition, might indicate an increase in cellular enzyme concentrations as response to elevated substrate concentrations, this adaptation did not abolish substrate inhibition of “*Ca*. N. kreftii”.

Lastly, the inhibitory effect of elevated ammonium concentrations on “*Ca*. N. kreftii” was verified in batch incubations. Parallel incubations were inoculated with biomass from the bioreactor system and amended with different amounts of substrate. Also in this setup, addition of elevated ammonium concentrations (>100 µM) decreased the observed maximum activity of ammonia oxidation, while nitrite oxidation rates continued to increase at higher nitrite concentrations (Fig. S6).

## Discussion

Recent metagenomic studies have demonstrated the abundance of comammox *Nitrospira* in numerous natural and engineered ecosystems, hinting at their crucial role within the biogeochemical nitrogen cycle [12]. However, the ecological niche of these novel organisms is still unclear. Theoretical kinetic modeling studies have predicted that comammox organisms would thrive in environments that select for low growth rates and high yields, as for instance encountered in biofilm-like systems under substrate-limited conditions [1, 2]. First physiological data of the pure culture *N. inopinata* substantiated these predictions, as this comammox *Nitrospira* was shown to have an extremely high ammonia affinity and growth yield, which is comparable to or even surpasses those of freshwater and terrestrial AOA [16, 63]. This indicates that in highly oligotrophic environments comammox *Nitrospira* could be one of the main drivers of nitrification. However, the limited availability of cultured representatives still hinders full appreciation of the unique comammox ecophysiology and thus additional cultures are urgently needed in order to fully understand their contribution to nitrification in the environment and their potential biotechnological applicability.

Here, a novel comammox *Nitrospira* species was highly enriched in a continuous substrate-limited bioreactor system. The enrichment culture performed complete nitrification without transient nitrite accumulation (Fig. S1). Metagenomic sequencing after 17 months of bioreactor operation revealed the presence of three canonical nitrite-oxidizing and one clade A comammox *Nitrospira* species (Fig. 2). Additional long-read sequencing facilitated the reconstruction of the complete genome of this comammox *Nitrospira*, which, based on pairwise ANI comparisons (Fig. S3) and phylogenetic distance (Fig. 2), forms a novel species tentatively named “*Ca*. N. kreftii”. Notably, this genome represents only the second complete genome available for comammox *Nitrospira*.

Genome analysis indicated high metabolic overlap with the phylogenetically closely related “*Ca*. N. nitrificans”, with which it also shares the highest genome identity (77% ANI). Like all comammox *Nitrospira*, they share the enzymatic machineries required for energy conservation by ammonia and nitrite oxidation. While the complexes of the respiratory chain including the periplasmic NXR are conserved among all *Nitrospira* [13], the key enzymes for ammonia and hydroxylamine oxidation are confined to comammox *Nitrospira* and have highest similarity to the respective enzymes in betaproteobacterial AOB [3, 4, 54]. For these it has recently been proposed that nitric oxide (NO) is an obligate intermediate of the ammonia oxidation process [64]. In this revised model, NO is produced by HAO and subsequently oxidized to nitrite abiotically or, more likely, enzymatically. One of the best candidates for NO oxidation is the NO-forming nitrite reductase NirK, which would operate in reverse during aerobic ammonia oxidation [64]. NirK is conserved in all *Nitrospira* including “*Ca*. N. kreftii”, but its function in the ammonia oxidation pathway remains to be verified.

The co-occurrence of canonical nitrite-oxidizing and comammox *Nitrospira* in the enrichment culture (Figs. 2 and S2) indicates a functional relationship between the two microorganisms in the system. Despite the fact that nitrite remained always below the detection limit (<5 µM) in the bioreactor, previous studies on *N. inopinata* have shown the transient accumulation of nitrite in comammox batch cultures [16]. Thus, comammox *Nitrospira* might always excrete some nitrite during ammonia oxidation, which in mixed culture systems might immediately be consumed by canonical *Nitrospira* with higher nitrite affinities [16]. This would explain the presence of canonical *Nitrospira* in the enrichment and indicate an unexpected potential interplay between the two functional types of *Nitrospira* similar to the symbiotic interactions between canonical AOB and NOB [65], with nitrite-oxidizing *Nitrospira* relying on leakage of nitrite from comammox *Nitrospira*.

Besides canonical *Nitrospira*, metagenomic sequencing furthermore indicated the presence of a complex microbial community, consisting mainly of potential heterotrophic microorganisms. Thus, despite the high degree of enrichment of “*Ca*. N. kreftii” achieved, a combination of physical separation and traditional microbiological techniques appears necessary to obtain a pure culture from the bioreactor’s biomass. Several protocols, including label-free cell sorting [66, 67], optical tweezers [68] and very recently an automated Raman-based microfluidics platform [69] could assist in the future isolation of “*Ca*. N. kreftii”. However, while pure cultures are of undoubtful importance for a thorough physiological characterization of an organism, also enrichment cultures can provide invaluable insights into their ecophysiology.

**Fig. 8:**
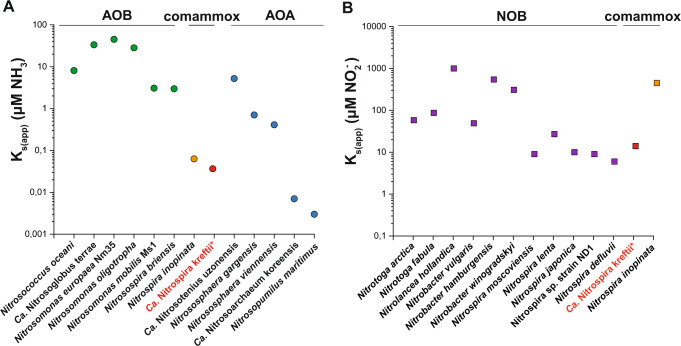
Comparison of the ammonia and nitrite affinities between canonical and complete nitrifying microorganisms. Apparent affinity constants for (**a**) ammonia and (**b**) nitrite of the “*Ca*. N. kreftii” enrichment culture (red symbols) in comparison to the reported values of *N. inopinata* (orange) and canonical AOA (blue), AOB (green) and NOB (purple) [16, 17, 72, 76–84]. When ammonia affinity values were not given in the respective studies, these were calculated from the reported total ammonium concentrations, pH and temperature provided. The asterisk indicates that the highly enriched “*Ca*. N. kreftii” culture contains also canonical, nitrite-oxidizing *Nitrospira*.

When we investigated the ammonia oxidation kinetics of our “*Ca*. N. kreftii” enrichment, we determined a very high ammonia affinity (mean K_m(app)_ ≈ 0.040 ± 0.01 µM NH_3_). However, this value must be considered as a conservative approximation, as diffusion limitations due to the flocculation of the biomass (average floc size 5.5 ± 10.4 µm^2^) are expected to have caused an underestimation of the substrate affinity. Correspondingly, when performed with less aggregated biomass (average floc size 3.0 ± 5.3 µm^2^), a higher substrate affinity was measured (K_m(app)_ ≈ 0.033 ± 0.012 µM NH_3_), and the opposite was observed an experiment with larger flocs (average floc size 8.4 ± 12.7 µm^2^, K_m(app)_ ≈ 0.051 ± 0.015 µM NH_3_; Fig. 4). These values are very similar to the reported ammonia affinity of *N. inopinata*, which however appears mostly in small cell aggregates and as planktonic cells [16], and confirm that comammox *Nitrospira* exhibit a substrate affinity orders of magnitude higher than most characterized AOB and even one order higher than many non-marine AOA (Fig. 8a). The high ammonia affinity determined for “*Ca*. N. kreftii” agrees well with previous theoretical predictions of the comammox ecophysiology [1, 2] and further verifies an adaptation of comammox bacteria to extremely oligotrophic environments [16].

Surprisingly, already very low ammonium concentrations (>25 µM) were found to partly inhibit ammonia oxidation by the “*Ca*. N. kreftii” enrichment. Although ammonium inhibition was not observed for *N. inopinata* [16], ammonium-sensitive AOA [17, 70] as well as canonical AOB affiliated with the genus *Nitrosomonas* [71–73] have been isolated previously, which however were only inhibited by ammonia concentrations in the low mM range. Moreover, ammonium-induced inhibition of nitrifying microorganisms in activated sludge and soil has been described as well [74, 75]. The inhibition of these ammonia-oxidizing microorganisms is thought to be a consequence of their adaptation to substrate-limited environments, or, alternatively, to be caused by a sensitivity to the toxic effects of free ammonia itself or to intermediates of the ammonia oxidation pathway [72, 75]. However, it was not possible to adapt the “*Ca*. N. kreftii” enrichment culture, as even after pre-incubation at higher ammonium concentrations (1 mM) for one month we still observed an inhibitory effect of ammonium concentrations >20–25 µM (Fig. 6). Moreover, in batch incubations with biomass from the enrichment culture, a lower ammonium oxidation rate was observed in the presence of >100 µM ammonium (Fig. S6), suggesting that this adaptation of “*Ca*. N. kreftii” to extremely low substrate concentrations was independent of the method used to study its ammonia oxidation kinetics and could not be attributed to continuous culturing under substrate-limited conditions. However, if there was any influence on the observed ammonia-oxidation kinetics from any heterotrophic microorganism still present in the enrichment culture will require further investigation, for instance when a pure culture of “*Ca*. N. kreftii” is obtained.

Nitrite oxidation in the “*Ca*. N. kreftii” enrichment followed canonical Michaelis–Menten kinetics and a substrate affinity consistent with canonical nitrite-oxidizing *Nitrospira* was obtained (mean K_m(app)_ = 12.5 ± 4.0 µM NO_2_^−^, *n* = 3; average floc size 4.4 ± 6.0 µm^2^; Fig. 5). As this value was determined in a system containing comammox and canonical nitrite-oxidizing *Nitrospira*, this represents the combined affinity of the two functionally distinct *Nitrospira* types. However, the low relative abundance of canonical nitrite-oxidizing *Nitrospira* at the time these experiments were conducted (3.1% of the total *Nitrospira* population; Fig. S2) suggests that also “*Ca*. N. kreftii” exhibits this high nitrite affinity, which is in stark contrast to *N. inopinata* (K_m(app)_ = 449.2 ± 65.8 µM NO_2_^−^) [16]. These physiological differences between comammox species emphasize the need for the investigation of several representatives of a microbial guild in order to obtain a complete picture of its ecophysiological potential.

In conclusion, the obtained enrichment culture enabled the genomic and physiological characterization of the novel comammox species “*Ca*. N. kreftii”. While there were only few metabolic differences predicted by genomic analyses compared to other clade A comammox *Nitrospira*, clear deviations were observed to *N. inopinata* regarding their ammonia and nitrite oxidation kinetics. The apparently higher substrate affinities of “*Ca*. N. kreftii” for ammonia compared to canonical AOB and many terrestrial AOA, and to nitrite compared to *N. inopinata*, indicate a physiological advantage in highly oligotrophic environments. Furthermore, the observed inhibition by ammonium implies differences in substrate tolerance of comammox *Nitrospira* that could play a crucial role in their interspecies competition and ecological niche partitioning. These novel insights into the physiology of comammox *Nitrospira* further expand our understanding of these unique microorganisms and can have significant implications on process design for their biotechnological application.

### Taxonomic consideration of “Candidatus Nitrospira kreftii” sp. nov

N.L. gen. n. kreftii, of Kreft, in honor of Jan-Ulrich Kreft, a German computational biologist, for his leading contribution to the theoretical prediction of comammox bacteria. Phylogenetically affiliated with sublineage II of the genus *Nitrospira*. Belongs to comammox clade A; capable of complete nitrification.

## Supplementary information

Supplemental Material

Dataset S1

Dataset S2

Dataset S3

## Data Availability

Sequencing data obtained in this study have been deposited in the National Center for Biotechnology Information (NCBI) database under Bioproject accession number PRJNA575653.

## References

[1] Costa E, Pérez J, Kreft J-U (2006). Why is metabolic labour divided in nitrification?. Trends Microbiol.

[2] van de Leemput IA, Veraart AJ, Dakos V, de Klein JJM, Strous M, Scheffer M (2011). Predicting microbial nitrogen pathways from basic principles. Environ Microbiol.

[3] van Kessel MAHJ, Speth DR, Albertsen M, Nielsen PH, Op den Camp HJM, Kartal B (2015). Complete nitrification by a single microorganism. Nature.

[4] Daims H, Lebedeva EV, Pjevac P, Han P, Herbold C, Albertsen M (2015). Complete nitrification by *Nitrospira* bacteria. Nature.

[5] Daims H, Lücker S, Wagner M (2016). A new perspective on microbes formerly known as nitrite-oxidizing bacteria. Trends Microbiol.

[6] Pjevac P, Schauberger C, Poghosyan L, Herbold CW, van Kessel MAHJ, Daebeler A (2017). *AmoA*-targeted polymerase chain reaction primers for the specific detection and quantification of comammox *Nitrospira* in the environment. Front Microbiol.

[7] Palomo A, Jane Fowler S, Gülay A, Rasmussen S, Sicheritz-Ponten T, Smets BF (2016). Metagenomic analysis of rapid gravity sand filter microbial communities suggests novel physiology of *Nitrospira* spp. ISME J.

[8] Pinto AJ, Marcus DN, Ijaz UZ, Bautista-de Lose Santos QM, Dick GJ, Raskin L (2016). Metagenomic evidence for the presence of comammox *Nitrospira*-like bacteria in a drinking water system. mSphere.

[9] Bartelme R, McLellan S, Newton R (2017). Freshwater recirculating aquaculture system operations drive biofilter bacterial community shifts around a stable nitrifying consortium of ammonia-oxidizing archaea and comammox *Nitrospira*. Front Microbiol.

[10] Wang Y, Ma L, Mao Y, Jiang X, Xia Y, Yu K (2017). Comammox in drinking water systems. Water Res.

[11] Orellana LH, Chee-Sanford JC, Sanford RA, Löffler FE, Konstantinidis KT (2018). Year-round shotgun metagenomes reveal stable microbial communities in agricultural soils and novel ammonia oxidizers responding to fertilization. Appl Environ Microbiol.

[12] Xia F, Wang J-G, Zhu T, Zou B, Rhee S-K, Quan Z-X (2018). Ubiquity and diversity of complete ammonia oxidizers (comammox). Appl Environ Microbiol.

[13] Koch H, van Kessel MAHJ, Lücker S (2018). Complete nitrification: insights into the ecophysiology of comammox *Nitrospira*. Appl Microbiol Biotechnol.

[14] Hu H-W, He J-Z (2017). Comammox—a newly discovered nitrification process in the terrestrial nitrogen cycle. J Soils Sediment.

[15] Fowler SJ, Palomo A, Dechesne A, Mines PD, Smets BF (2018). Comammox *Nitrospira* are abundant ammonia oxidizers in diverse groundwater-fed rapid sand filter communities. Environ Microbiol.

[16] Kits KD, Sedlacek CJ, Lebedeva EV, Han P, Bulaev A, Pjevac P (2017). Kinetic analysis of a complete nitrifier reveals an oligotrophic lifestyle. Nature.

[17] Martens-Habbena W, Berube PM, Urakawa H, de la Torre JR, Stahl DA (2009). Ammonia oxidation kinetics determine niche separation of nitrifying Archaea and Bacteria. Nature.

[18] Spieck E, Lipski A (2011). Cultivation, growth physiology, and chemotaxonomy of nitrite-oxidizing bacteria. Methods Enzymol.

[19] Taylor S, Ninjoor V, Dowd DM, Tappel AL (1974). Cathepsin B2 measurement by sensitive fluorometric ammonia analysis. Anal Biochem.

[20] Griess P (1879). Bemerkungen zu der Abhandlung der HH. Weselsky und Benedikt „Ueber einige Azoverbindungen”.. Ber Dtsch Chem Ges..

[21] Daims H, Lücker S, Wagner M (2006). *daime*, a novel image analysis program for microbial ecology and biofilm research. Environ Microbiol.

[22] Daims H, Wagner M (2007). Quantification of uncultured microorganisms by fluorescence microscopy and digital image analysis. Appl Microbiol Biotechnol.

[23] Zhou J, Bruns MA, Tiedje JM (1996). DNA recovery from soils of diverse composition. Appl Environ Microbiol.

[24] Nurk S, Meleshko D, Korobeynikov A, Pevzner PA (2017). metaSPAdes: a new versatile metagenomic assembler. Genome Res.

[25] Li H, Durbin R (2010). Fast and accurate long-read alignment with Burrows-Wheeler transform. Bioinformatics.

[26] Li H, Handsaker B, Wysoker A, Fennell T, Ruan J, Homer N (2009). The sequence alignment/map format and SAMtools. Bioinformatics.

[27] Graham ED, Heidelberg JF, Tully BJ (2017). BinSanity: unsupervised clustering of environmental microbial assemblies using coverage and affinity propagation. PeerJ.

[28] Lu YY, Chen T, Fuhrman JA, Sun F (2017). COCACOLA: binning metagenomic contigs using sequence COmposition, read CoverAge, CO-alignment and paired-end read LinkAge. Bioinformatics.

[29] Alneberg J, Bjarnason BS, de Bruijn I, Schirmer M, Quick J, Ijaz UZ (2014). Binning metagenomic contigs by coverage and composition. Nat Methods.

[30] Wu YW, Simmons BA, Singer SW (2016). MaxBin 2.0: an automated binning algorithm to recover genomes from multiple metagenomic datasets. Bioinformatics.

[31] Kang DD, Froula J, Egan R, Wang Z (2015). MetaBAT, an efficient tool for accurately reconstructing single genomes from complex microbial communities. PeerJ.

[32] Sieber CMK, Probst AJ, Sharrar A, Thomas BC, Hess M, Tringe SG (2018). Recovery of genomes from metagenomes via a dereplication, aggregation and scoring strategy. Nat Microbiol.

[33] Parks DH, Imelfort M, Skennerton CT, Hugenholtz P, Tyson GW (2015). CheckM: assessing the quality of microbial genomes recovered from isolates, single cells, and metagenomes. Genome Res.

[34] Parks DH, Chuvochina M, Waite DW, Rinke C, Skarshewski A, Chaumeil PA (2018). A standardized bacterial taxonomy based on genome phylogeny substantially revises the tree of life. Nat Biotechnol.

[35] Chaumeil P-A, Mussig AJ, Hugenholtz P, Parks DH (2019). GTDB-Tk: a toolkit to classify genomes with the Genome Taxonomy Database. Bioinformatics.

[36] Koren S, Walenz BP, Berlin K, Miller JR, Bergman NH, Phillippy AM (2017). Canu: scalable and accurate long-read assembly via adaptive k-mer weighting and repeat separation. Genome Res.

[37] Li H (2018). Minimap2: pairwise alignment for nucleotide sequences. Bioinformatics.

[38] Vaser R, Sović I, Nagarajan N, Šikić M (2017). Fast and accurate de novo genome assembly from long uncorrected reads. Genome Res.

[39] Na S-I, Kim YO, Yoon S-H, Ha S-m, Baek I, Chun J (2018). UBCG: Up-to-date bacterial core gene set and pipeline for phylogenomic tree reconstruction. J Microbiol.

[40] Stamatakis A (2014). RAxML version 8: a tool for phylogenetic analysis and post-analysis of large phylogenies. Bioinformatics.

[41] Miller MA, Pfeiffer W, Schwartz T, editors. Creating the CIPRES science gateway for inference of large phylogenetic trees. 2010 Gateway Computing Environments Workshop (GCE); 2010.

[42] Yoon S-H, Ha S-m, Lim J, Kwon S, Chun J (2017). A large-scale evaluation of algorithms to calculate average nucleotide identity. Antonie van Leeuwenhoek..

[43] Seemann T (2014). Prokka: rapid prokaryotic genome annotation. Bioinformatics.

[44] Vallenet D, Labarre L, Rouy Z, Barbe V, Bocs S, Cruveiller S (2006). MaGe: a microbial genome annotation system supported by synteny results. Nucleic Acids Res.

[45] Krzywinski M, Schein J, Birol I, Connors J, Gascoyne R, Horsman D (2009). Circos: an information aesthetic for comparative genomics. Genome Res.

[46] Schindelin J, Arganda-Carreras I, Frise E, Kaynig V, Longair M, Pietzsch T (2012). Fiji: an open-source platform for biological-image analysis. Nat Methods.

[47] Kassambara A. rstatix: pipe-friendly framework for basic statistical tests. 2020. https://rpkgs.datanovia.com/rstatix/.

[48] R Core Team. R: a language and environment for statistical computing. 2019. https://www.R-project.org/.

[49] Richter M, Rosselló-Móra R (2009). Shifting the genomic gold standard for the prokaryotic species definition. Proc Natl Acad Sci USA.

[50] Sakoula D, Nowka B, Spieck E, Daims H, Lücker S (2018). The draft genome sequence of “*Nitrospira lenta*” strain BS10, a nitrite oxidizing bacterium isolated from activated sludge. Stand Genom Sci.

[51] Koch H, Lücker S, Albertsen M, Kitzinger K, Herbold C, Spieck E (2015). Expanded metabolic versatility of ubiquitous nitrite-oxidizing bacteria from the genus *Nitrospira*. Proc Natl Acad Sci USA.

[52] Lücker S, Wagner M, Maixner F, Pelletier E, Koch H, Vacherie B (2010). A *Nitrospira* metagenome illuminates the physiology and evolution of globally important nitrite-oxidizing bacteria. Proc Natl Acad Sci USA.

[53] Lawson CE, Lücker S (2018). Complete ammonia oxidation: an important control on nitrification in engineered ecosystems?. Curr Opin Biotechnol.

[54] Palomo A, Pedersen AG, Fowler SJ, Dechesne A, Sicheritz-Pontén T, Smets BF (2018). Comparative genomics sheds light on niche differentiation and the evolutionary history of comammox *Nitrospira*. ISME J.

[55] Dibrova DV, Galperin MY, Mulkidjanian AY (2010). Characterization of the N-ATPase, a distinct, laterally transferred Na^+^-translocating form of the bacterial F-type membrane ATPase. Bioinformatics.

[56] Soontharapirakkul K, Promden W, Yamada N, Kageyama H, Incharoensakdi A, Iwamoto-Kihara A (2011). Halotolerant cyanobacterium *Aphanothece halophytica* contains an Na^+^-dependent F_1_F_o_-ATP synthase with a potential role in salt-stress tolerance.. J Biol Chem..

[57] de Almeida NM, Wessels HJCT, de Graaf RM, Ferousi C, Jetten MSM, Keltjens JT (2016). Membrane-bound electron transport systems of an anammox bacterium: a complexome analysis. Biochimica et Biophysica Acta (BBA) - Bioenerg.

[58] Frank J, Lücker S, Vossen RHAM, Jetten MSM, Hall RJ, Op den Camp HJM (2018). Resolving the complete genome of *Kuenenia stuttgartiensis* from a membrane bioreactor enrichment using single-molecule real-time sequencing. Sci Rep.

[59] Daebeler A, Kitzinger K, Koch H, Herbold CW, Steinfeder M, Schwarz J (2020). Exploring the upper pH limits of nitrite oxidation: diversity, ecophysiology, and adaptive traits of haloalkalitolerant *Nitrospira*.. ISME J..

[60] Poghosyan L, Koch H, Lavy A, Frank J, van Kessel MAHJ, Jetten MSM (2019). Metagenomic recovery of two distinct comammox *Nitrospira* from the terrestrial subsurface. Environ Microbiol.

[61] Picioreanu C, Pérez J, van Loosdrecht MCM (2016). Impact of cell cluster size on apparent half-saturation coefficients for oxygen in nitrifying sludge and biofilms. Water Res.

[62] Okabe S, Kindaichi T, Ito T, Satoh H (2004). Analysis of size distribution and areal cell density of ammonia-oxidizing bacterial microcolonies in relation to substrate microprofiles in biofilms. Biotechnol Bioeng.

[63] Straka LL, Meinhardt KA, Bollmann A, Stahl DA, Winkler MH (2019). Affinity informs environmental cooperation between ammonia-oxidizing archaea (AOA) and anaerobic ammonia-oxidizing (Anammox) bacteria. ISME J.

[64] Caranto JD, Lancaster KM (2017). Nitric oxide is an obligate bacterial nitrification intermediate produced by hydroxylamine oxidoreductase. Proc Natl Acad Sci USA.

[65] Gruber-Dorninger C, Pester M, Kitzinger K, Savio DF, Loy A, Rattei T (2015). Functionally relevant diversity of closely related *Nitrospira* in activated sludge. ISME J.

[66] Fujitani H, Kumagai A, Ushiki N, Momiuchi K, Tsuneda S (2015). Selective isolation of ammonia-oxidizing bacteria from autotrophic nitrifying granules by applying cell-sorting and sub-culturing of microcolonies. Front Microbiol.

[67] Ushiki N, Fujitani H, Aoi Y, Tsuneda S (2013). Isolation of *Nitrospira* belonging to sublineage II from a wastewater treatment plant. Microbes Environ.

[68] Nowka B, Off S, Daims H, Spieck E (2015). Improved isolation strategies allowed the phenotypic differentiation of two *Nitrospira* strains from widespread phylogenetic lineages. FEMS Microbiol Ecol.

[69] Lee KS, Palatinszky M, Pereira FC, Nguyen J, Fernandez VI, Mueller AJ (2019). An automated Raman-based platform for the sorting of live cells by functional properties. Nat Microbiol.

[70] Hatzenpichler R, Lebedeva EV, Spieck E, Stoecker K, Richter A, Daims H (2008). A moderately thermophilic ammonia-oxidizing crenarchaeote from a hot spring. Proc Natl Acad Sci USA.

[71] Suwa Y, Imamura Y, Suzuki T, Tashiro T, Urushigawa Y (1994). Ammonia-oxidizing bacteria with different sensitivities to (NH_4_)_2_SO_4_ in activated sludges. Water Res.

[72] Bollmann A, Laanbroek HJ (2001). Continuous culture enrichments of ammonia-oxidizing bacteria at low ammonium concentrations. FEMS Microbiol Ecol.

[73] Suwa Y, Sumino T, Noto K (1997). Phylogenetic relationships of activated sludge isolates of ammonia oxidizers with different sensitivities to ammonium sulfate. J Gen Appl Microbiol.

[74] Keenan JD, Steiner RL, Fungaroli AA (1979). Substrate inhibition of nitrification. J Environ Sci Health Part A: Environ Sci Eng.

[75] Koper TE, Stark JM, Habteselassie MY, Norton JM (2010). Nitrification exhibits Haldane kinetics in an agricultural soil treated with ammonium sulfate or dairy-waste compost. FEMS Microbiol Ecol.

[76] Jung MY, Park SJ, Min D, Kim JS, Rijpstra WI, Sinninghe Damste JS (2011). Enrichment and characterization of an autotrophic ammonia-oxidizing archaeon of mesophilic crenarchaeal group I.1a from an agricultural soil. Appl Environ Microbiol.

[77] Thandar SM, Ushiki N, Fujitani H, Sekiguchi Y, Tsuneda S (2016). Ecophysiology and comparative genomics of *Nitrosomonas mobilis* Ms1 isolated from autotrophic nitrifying granules of wastewater treatment bioreactor. Front Microbiol.

[78] Hayatsu M, Tago K, Uchiyama I, Toyoda A, Wang Y, Shimomura Y (2017). An acid-tolerant ammonia-oxidizing γ-proteobacterium from soil. ISME J..

[79] Ward BB (1987). Kinetic studies on ammonia and methane oxidation by *Nitrosococcus oceanus*. Arch Microbiol.

[80] Nowka B, Daims H, Spieck E (2015). Comparison of oxidation kinetics of nitrite-oxidizing bacteria: nitrite availability as a key factor in niche differentiation. Appl Environ Microbiol.

[81] Ushiki N, Jinno M, Fujitani H, Suenaga T, Terada A, Tsuneda S (2017). Nitrite oxidation kinetics of two *Nitrospira* strains: the quest for competition and ecological niche differentiation. J Biosci Bioeng.

[82] Kitzinger K, Koch H, Lücker S, Sedlacek CJ, Herbold C, Schwarz J (2018). Characterization of the first “*Candidatus* Nitrotoga” isolate reveals metabolic versatility and separate evolution of widespread nitrite-oxidizing bacteria. mBio.

[83] Sorokin DY, Lücker S, Vejmelkova D, Kostrikina NA, Kleerebezem R, Rijpstra WI (2012). Nitrification expanded: discovery, physiology and genomics of a nitrite-oxidizing bacterium from the phylum *Chloroflexi*. ISME J.

[84] Groeneweg J, Sellner B, Tappe W (1994). Ammonia oxidation in *Nitrosomonas* at NH_3_ concentrations near K_m_: Effects of pH and temperature. Water Res.

