# Supplementary material for: Enrichment and physiological characterization of a novel comammox Nitrospira indicates ammonium inhibition of complete nitrification
Source: ISME J. 2020 Nov 13;15(4):1010–24. doi: 10.1038/s41396-020-00827-4 (PMC8115096; doi:10.1038/s41396-020-00827-4)
Supplement: Supplementary file 1 — Supplemental Material [file 41396_2020_827_MOESM1_ESM.docx]

**Supplementary Information for:**

**Enrichment and physiological characterization of a novel comammox *Nitrospira* indicates ammonium inhibition of complete nitrification**

Dimitra Sakoula^1,#,^*, Hanna Koch^1^, Jeroen Frank^1,2^, Mike SM Jetten^1,2^, Maartje AHJ van Kessel^1^, Sebastian Lücker^1,^*

^1^Department of Microbiology, IWWR, Radboud University, Heyendaalseweg 135, 6525 AJ Nijmegen, the Netherlands.

^2^Soehngen Institute of Anaerobic Microbiology, Radboud University, Heyendaalseweg 135, 6525 AJ Nijmegen, the Netherlands.

^#^Present address: Division of Microbial Ecology, Center for Microbiology and Environmental Systems Science, University of Vienna, Althanstraße 14, 1090, Vienna, Austria.

***Corresponding authors:**

Dimitra Sakoula, Division of Microbial Ecology, Center for Microbiology and Environmental Systems Science, University of Vienna, Althanstraße 14, 1090, Vienna, Austria; mail: [dimitra.sakoula@univie.ac.at](mailto:dimitra.sakoula@univie.ac.at)

Sebastian Lücker, Department of Microbiology, IWWR, Radboud University, Heyendaalseweg 135, 6525 AJ Nijmegen, the Netherlands; mail: [s.luecker@science.ru.nl](mailto:s.luecker@science.ru.nl).

**Supplemental figures and tables**


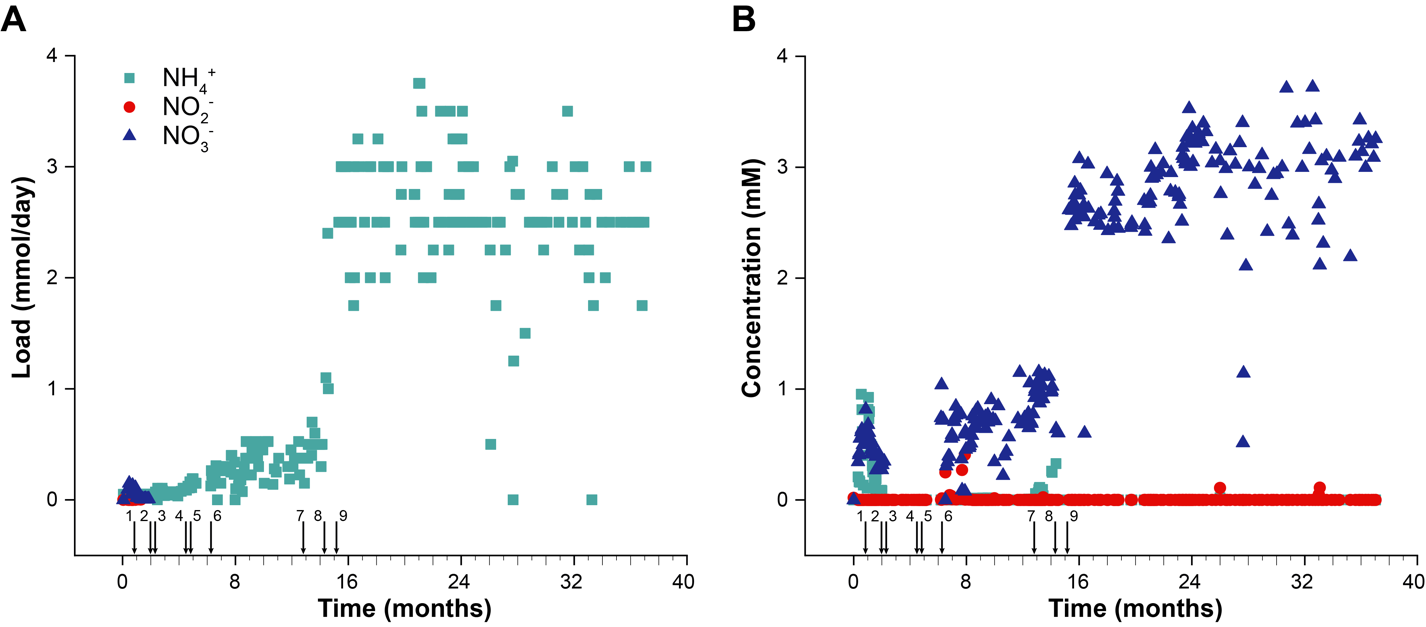


**Figure S1.** Substrate loading (A) and apparent substrate concentrations (B) in the bioreactor system during the enrichment period. Symbols indicate ammonium (squares), nitrite (circles) and nitrate (triangles). Numbered arrows indicate the time points that substrate concentrations in the medium supplied to the system were manipulated; 1: 250/20/500 µM NH_4_Cl/NaNO_2_/NaNO_3_ (day 27), 2: 250 µM NH_4_Cl (day 60), 3: 350 µM NH_4_Cl (day 69), 4: 450 µM NH_4_Cl (day 139), 5: 500 µM NH_4_Cl (day 145), 6: 750 µM NH_4_Cl (day 186), 7: 1 mM NH_4_Cl (day 388), 8: 2 mM NH_4_Cl (day 426), and 9: 2.5 mM NH_4_Cl (day 453).


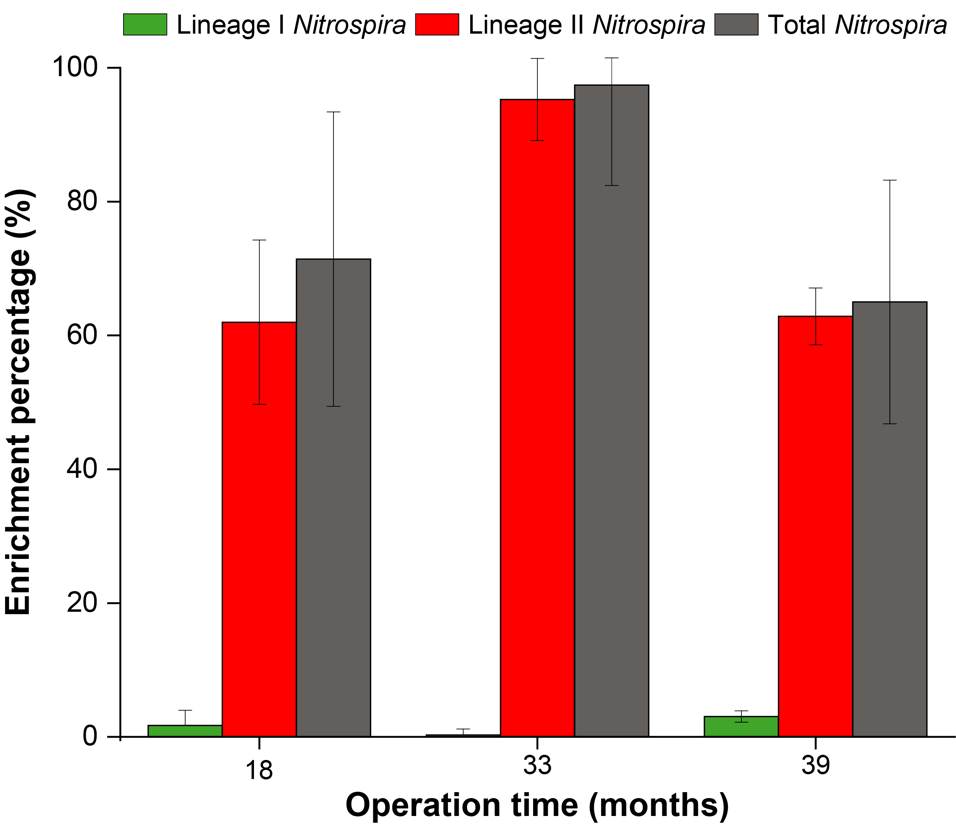


**Figure S2**. Relative abundance of sublineage I and II *Nitrospira* bacteria in the bioreactor system over the enrichment period. Sublineage abundances were normalized in relation to the relative abundance of the total *Nitrospira* population in the enrichment culture.

**
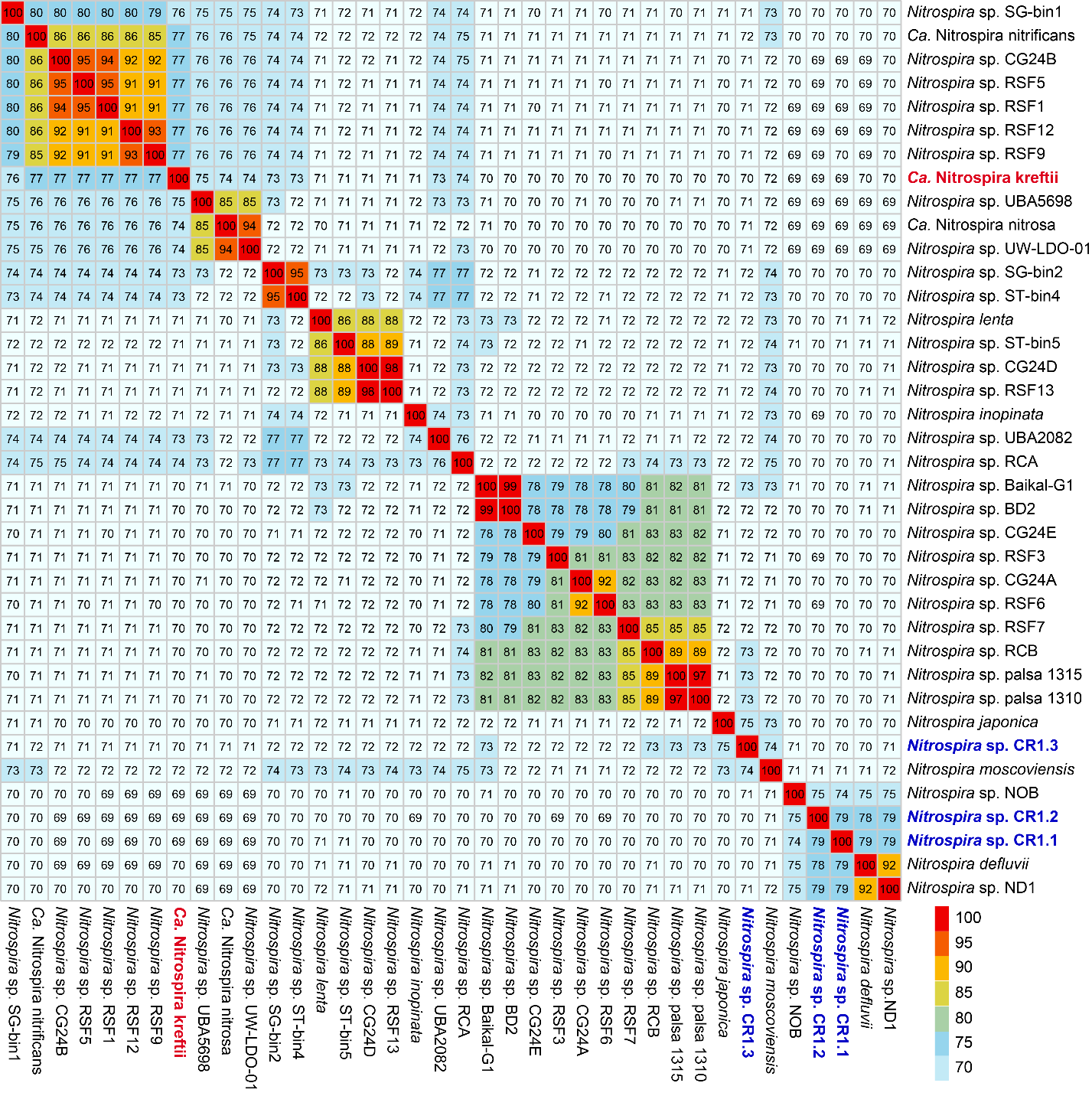
**

**Figure S3.** Genome similarity heatmap showing the pairwise ANI comparisons between the *Nitrospira* MAGs obtained in this study (in bold) and publicly available high-quality genomes of sublineage I and II *Nitrospira* species.

**
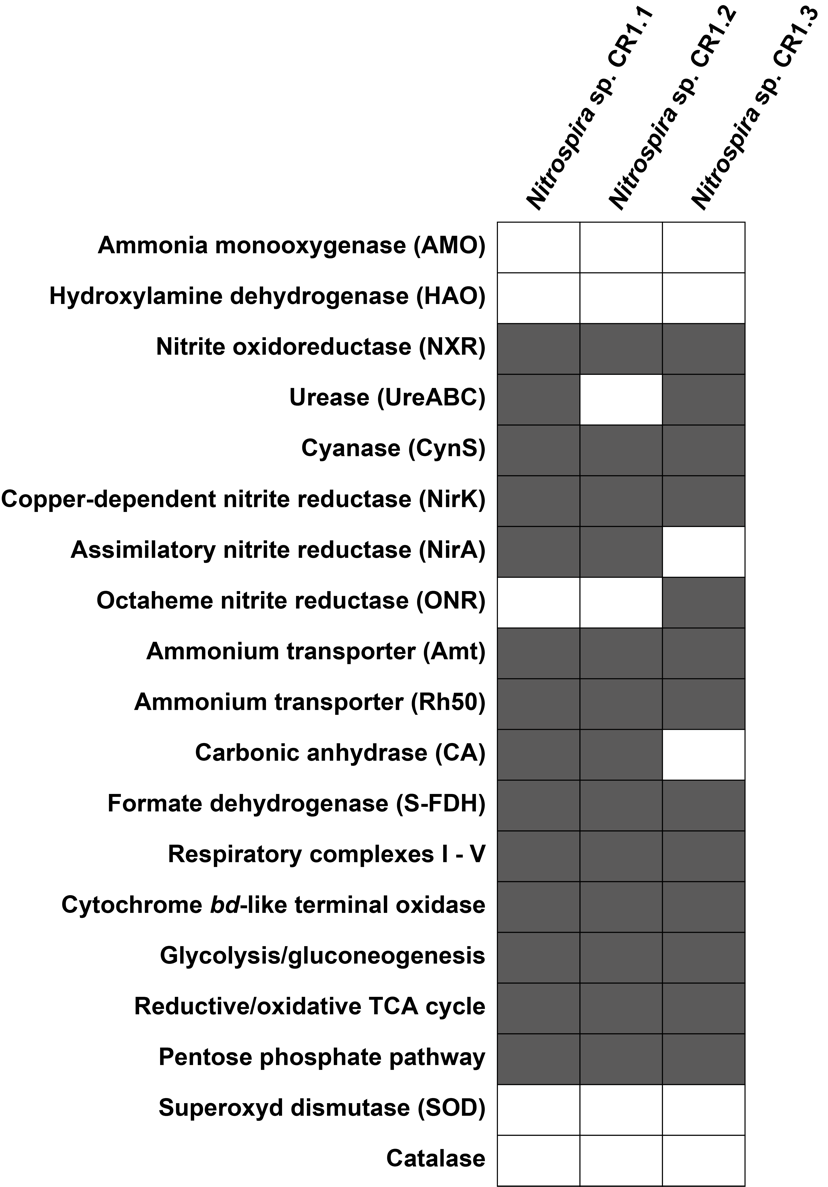
**

**Figure S4.** Distribution pattern of key metabolic features involved in nitrogen and alternative energy metabolisms in the canonical nitrite-oxidizing *Nitrospira* MAGs retrieved in this study. Dark grey and white indicate presence and absence of the respective genes, respectively.

**
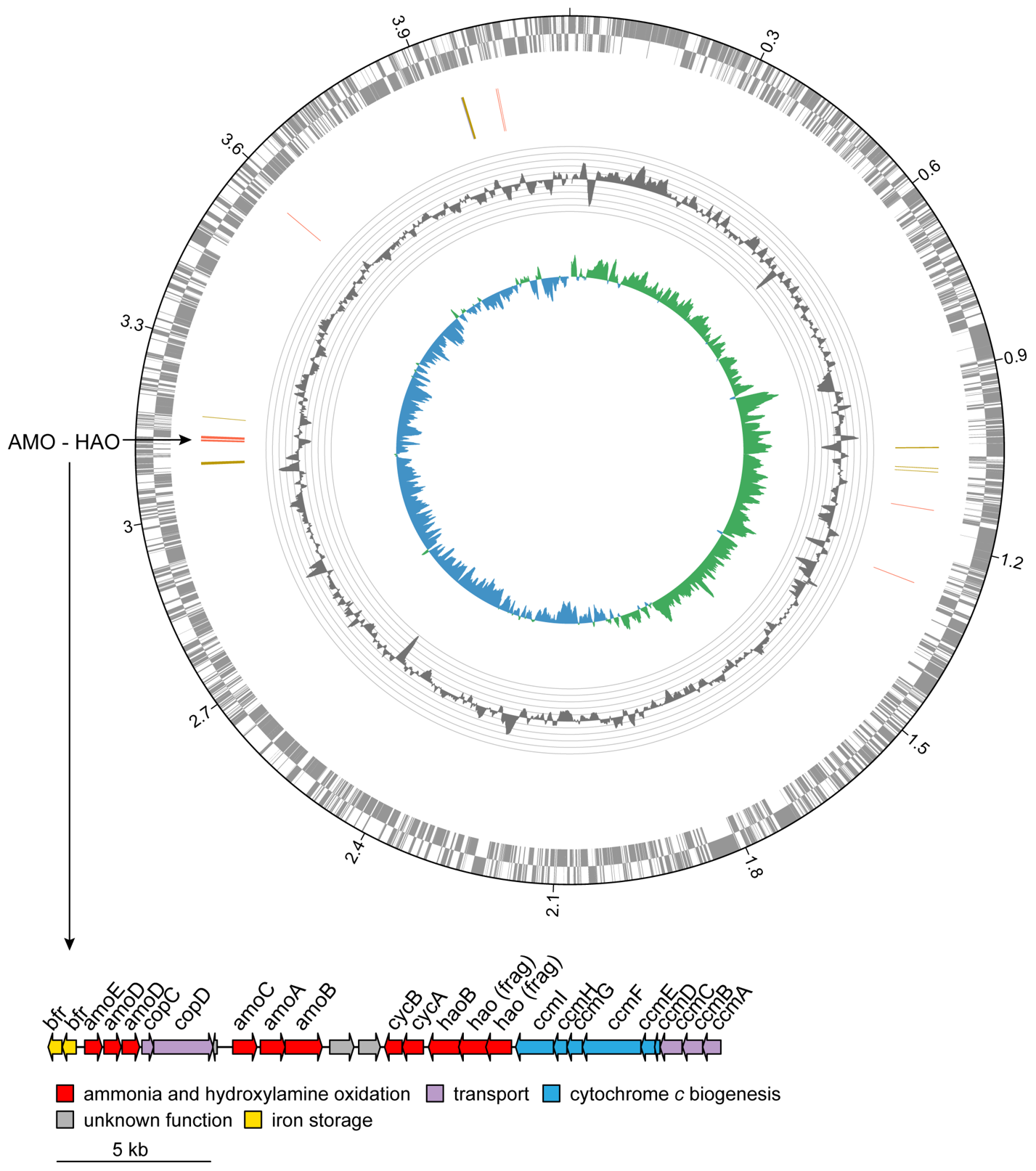
**

**Figure S5.** Circular representation of the “*Ca.* N. kreftii” chromosome. From outside to inside the rings display: (1) and (2) predicted coding sequences on forward and reverse strand, respectively, (3) Genes involved in ammonia and nitrite oxidation. Red: *amoABC*, *haoAB* and *cycAB*; orange: *nxrABC*. (4) Local GC bias and (5) GC skew (green: positive, blue: negative).

**
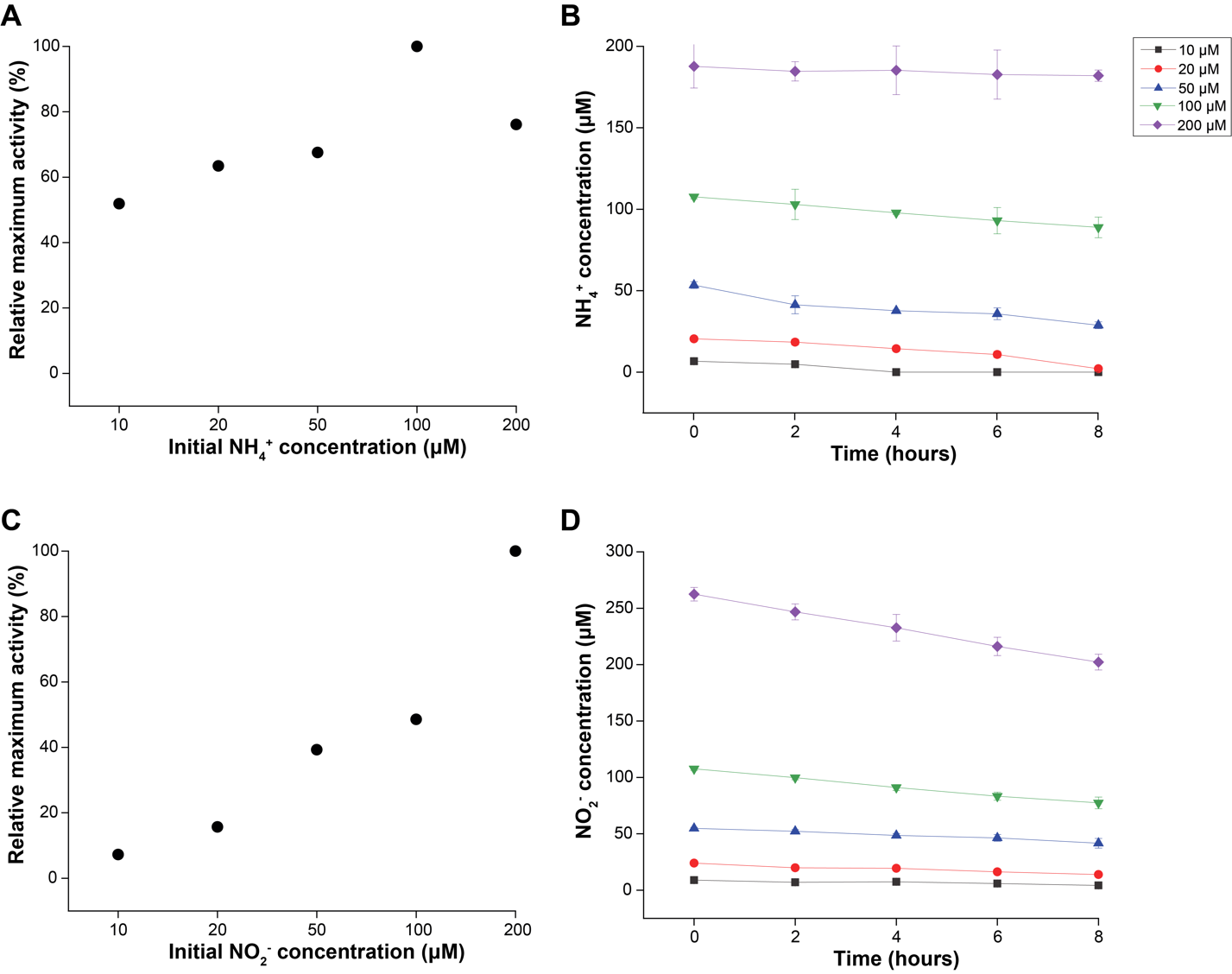
**

**Figure S6.** Ammonium (A, B) and nitrite oxidation (C, D) activity of batch incubations inoculated with the “*Ca*. N. kreftii” enrichment culture. Relative maximum (A) ammonium and (C) nitrite oxidation rates as recorded at the substrate concentrations shown in (B) and (D) and calculated in relation to the maximum oxidation rate observed during the respective experiment. Symbols and error bars represent averages and standard deviations of three biological replicates, respectively.

**Table S1.** Specifications of the FISH probes used in this study.

| **Probe** | **Target** | **FA %^a^** | **Sequence (5’🡪3’)** | **Reference** |
| --- | --- | --- | --- | --- |
| **Ntspa712** | *Nitrospira* phylum  (most members) | 35 | CGCCTTCGCCACCGGCCTTCC | (1) |
| **Comp-Ntspa712** | Competitor to Ntspa712 | - | CGCCTTCGCCACCGGTGTTCC | (1) |
| **Ntspa662** | genus *Nitrospira* | 35 | GGAATTCCGCGCTCCTCT | (1) |
| **Comp-Ntspa662** | Competitor to Ntspa662 | - | GGAATTCCGCTCTCCTCT | (1) |
| **Ntspa1431** | Sublineage I of the genus *Nitrospira* | 35 | TTGGCTTGGGCGACTTCA | (2) |
| **Ntspa1151** | Sublineage II of the genus *Nitrospira* | 35-40 | TTCTCCTGGGCAGTCTCT CC | (2) |
| **EUB338 (Bact338)** | Most bacteria | 0-50 | GCTGCCTCCCGTAGGAGT | (3) |
| **EUB338 II (SBACT P 338)** | *Planctomycetales* | 0-50 | GCAGCCACCCGTAGGTGT | (4) |
| **EUB338 III (SBACT V 338)** | *Verrucomicrobiales* | 0-50 | GCTGCCACCCGTAGGTGT | (4) |

^a^Concentration of formamide (FA) in the hybridization buffer

**Table S2.** Enrichment of the bioreactor’s biomass in *Nitrospira* bacteria over the total enrichment period determined by quantitative FISH.

| **Operation time (months)** | **Enrichment (%)** | **S.E. (±)** |
| --- | --- | --- |
| 0 | 5.4 | 8.9 |
| 8 | 53 | 17.9 |
| 11 | 71.3 | 25.5 |
| 14 | 73.7 | 14.7 |
| 17 | 57 | 29.5 |
| 18 | 64.8 | 15 |
| 19 | 83.5 | 14.9 |
| 24 | 65.3 | 18.9 |
| 26 | 85.1 | 6.5 |
| 27 | 90.5 | 7.6 |
| 28 | 86.1 | 10 |
| 33 | 71.7 | 8 |
| 35 | 55.9 | 17.5 |
| 39 | 71.9 | 18.2 |

**Dataset S1 (separate file).** Overview of the medium- and high-quality metagenome-assembled genomes (MAGs; completeness ≥75%, contamination ≤10%) obtained from the enrichment culture after 17 months of enrichment.

**Dataset S2 (separate file).** Overview of the medium- and high-quality metagenome-assembled genomes (MAGs; completeness ≥75%, contamination ≤10%) obtained from the enrichment culture after 39 months of enrichment.

**Dataset S3 (separate file).** **“***Ca.* Nitrospira kreftii” proteins with predicted functions in key metabolic pathways.

**SI References**

1. Daims H, Nielsen JL, Nielsen PH, Schleifer KH, Wagner M. In situ characterization of Nitrospira-like nitrite-oxidizing bacteria active in wastewater treatment plants. Applied and environmental microbiology. 2001;67(11):5273-84.

2. Maixner F, Noguera DR, Anneser B, Stoecker K, Wegl G, Wagner M, et al. Nitrite concentration influences the population structure of Nitrospira-like bacteria. Environmental Microbiology. 2006;8(8):1487-95.

3. Amann RI, Binder BJ, Olson RJ, Chisholm SW, Devereux R, Stahl DA. Combination of 16S rRNA-targeted oligonucleotide probes with flow cytometry for analyzing mixed microbial populations. Applied and Environmental Microbiology. 1990;56(6):1919-25.

4. Daims H, Brühl A, Amann R, Schleifer K-H, Wagner M. The Domain-specific Probe EUB338 is Insufficient for the Detection of all Bacteria: Development and Evaluation of a more Comprehensive Probe Set. Systematic and Applied Microbiology. 1999;22(3):434-44.
